# Something Old, Something New, Something Borrowed… About the Placenta

**DOI:** 10.3390/epigenomes10010005

**Published:** 2026-01-19

**Authors:** Nadezhda Milova, Maria Nikolova, Angel Yordanov, Antoan Milov, Stoilka Mandadzhieva

**Affiliations:** 1 Center for Women’s Health, 4000 Plovdiv, Bulgaria; nadezhda.milova@cwh-bg.com (N.M.); antoan.milov@cwh-bg.com (A.M.); 2 Faculty of Medicine, Medical University of Plovdiv, 4000 Plovdiv, Bulgaria; 3 Department of Gynecological Oncology, Medical University Pleven, 5800 Pleven, Bulgaria; angel.jordanov@gmail.com; 4 Pathophysiology Department, Medical University of Plovdiv, 4000 Plovdiv, Bulgaria; smandadzhieva@pathophysiology.info

**Keywords:** miRNA, piRNA, placenta, preeclampsia, endometrial receptivity

## Abstract

The connection between the mother and the child has been considered one of the strongest bonds in nature. Though there are numerous factors that can influence the establishment of pregnancy, in its essence, three are considered major: a good quality embryo, a receptive endometrium, and successful cross-talk between them. The placenta, which derives from the trophoblast of the embryo, develops when a successful implantation occurs. It is an ephemeral organ through which the turnover of nutrients, gases, and waste molecules is realized. It serves as a barrier and can provide the embryo with immune factors. Placental disorders are observed in some rare but life-threatening obstetric conditions like preeclampsia (PE), fetal growth restriction (FGR), gestational trophoblastic diseases (GTDs), and gestational diabetes mellitus (GDM). The etiology and pathogenesis of some are still partially enigmatic. Our attention in this review was driven by the participation of small RNA molecules—miRNAs and piRNAs—as potential epigenetic modulators of genes that play a pivotal role in placental functioning. In this study, we analyze the influence of these epigenetic factors on the mechanisms of the development of preeclampsia. The molecular approach for understanding placental disorders may help new diagnostic and therapeutic solutions to be found.

## 1. Introduction

The title that we have chosen comes from a traditional English wedding rhyme and is an invocation for a strong union. The bond between the uterus and the placenta is essential for the normal development of pregnancy. The *placenta*, from the Greek πλακόεντα, with the meaning of “flat cake” and named after its round, flat appearance in humans, is an ephemeral organ that develops from the trophectoderm of the implanting blastocyst [1,2]. It plays a crucial role for the embryo as it facilitates the exchange of nutrients, gases, and waste products between the actually separate maternal and fetal circulations. It has endocrine function, and the hormones it produces regulate both maternal and fetal physiological processes throughout the pregnancy. The placenta is connected to the fetus via the umbilical cord, and the opposite side is connected to the maternal uterus. In humans, after the birth of the child, part of the maternal endometrium is expelled with the placenta when it is delivered from the uterus. All placental mammals start their development with the crucial participation of the placenta, but some marsupials and some non-mammals are also placental species [3]. The placenta has emerged in evolution independently multiple times, starting in fish and appearing in some reptiles as well [4,5].

The mammal placenta first occurred about 100 million years ago. Mammalian placentas have a common function, but they differ morphologically and functionally between mammalian groups [6]. They vary in gross and microscopic views, as well as in their function; for example, not all can supply the fetus with maternal immunoglobulins [6]. It has been hypothesized that the highly species-specific transposable elements [TEs], in particular the endogenous retroviruses, are the major drivers of the fast evolution of the placenta. An example of TE-derived genes is syncytin, which is essential for cell-to-cell fusion and the formation of the multinucleated syncytiotrophoblast layer. It has been presumed that it probably facilitated the transition from egg laying to live birth [6,7,8].

The placenta is a vital organ for the establishment and support of pregnancy, and any disturbance in its function can critically influence the outcome of pregnancy. Among other disorders like FGR, GTD, and GDM, PE outstands as the most life-threatening one for both the mother and the child.

Epigenetics is today considered to profoundly influence life and longevity, from the very moment of the occurrence of life, throughout the lifespan of the individual, until the life ends [9]. The epigenetic control of switched-on and off genes leads to the alteration of gene expression with an impact on every individual developmental trait. In this study, we aim to analyze some of the epigenetic factors that influence the development and function of the placenta and their influence on the mechanisms of the development of preeclampsia, especially of two non-coding RNA molecules—namely miRNAs and piRNAs [10].

## 2. Physiology of the Placenta

### 2.1. Conception and Implantation

Just after ovulation occurs, the released oocyte is gently taken over by the fallopian tube. There it remains in the ampullary part for 36–48 h, waiting to be fertilized by the spermatozoa, coming from the lower genital tract [2]. If conception occurs, then the newly formed zygote starts a long 5–6-day journey in the tube, advancing towards the uterine cavity. It enters the cavity as a free-floating blastocyst surrounded by zona pellucida, consisting of an inner cell mass with a trophectoderm hull. Rolling on the carpet of the endometrium, it hatches from the zona pellucida. Appositioning of the polar hatched blastocyst occurs at a putative site, probably orchestrated by some endometrial molecules, and cross-talk occurs between the embryo and the receptive endometrium. The blastocyst adheres to the endometrium, and the differentiation of trophoblast cells starts, into cytotrophoblast, an inner mass, and syncytiotrophoblast, the outer part. The syncytiotrophoblast invades the luminal epithelium, and complete implantation follows [2]. Macroscopically, at the moment of attachment, the trophoblast grows and forms a large syncytial cytoplasmic mass—a syncytiotrophoblast (STB). STBs form sheaths that, by lysing the underlying tissues, penetrate deep into the endometrium and pave the way for the blastocyst to sink. STBs first overcome the uterine epithelium and then the underlying decidua (loose connective tissue), and they finally destroy the walls of the capillaries. Thus, the blastocyst finds itself in direct contact with the mother’s blood, which freely pours out of the ruptured vessels into the spaces formed around it, called lacunae. Some of the trophoblast cells, the extravillous trophoblast cells, displace the cells of the endoglandular epithelium [11]. Implantation is considered complete upon the sinking of the blastocyst into the endometrium; the entrance site is closed by the regeneration of connective tissue and the growth of a new covering epithelium [11]. The first schematic description of implantation was made by Elizabeth Ramsey, who later depicted the changes in uterine arteries and veins during pregnancy [11]. Until recently, it was assumed that trophoblastic invasion in the process of human placentation was limited to entering the connective tissue of the uterus and the uterine spiral arteries. It was believed that the spiral arteries connect with the intervillous space of the placenta and thus ensure a supply of maternal blood to the fetus. In recent years, this conception has changed significantly. The invasion of the endoglandular trophoblast into the endometrial glands from the start of implantation creates the first nutrition for the fetus—the histiotrophic through the so-called “uterine milk” [12,13,14,15,16]. What follows is the invasion of the endovenous trophoblast into the uterine veins, which provides an outflow of fluids back into the maternal circulation [13,17,18]. The entry of the endolymphatic trophoblast into the lymphatic vessels of the uterus follows. Only then does the endoarterial trophoblast invade the spiral arteries, providing hemotropic nutrition, beginning in the second trimester of pregnancy [16].

The polarization of endometrial epithelial cells in the mid-secretory phase is considered to be an essential factor in the occurrence of implantation [19]. As the endometrium remodels in the mid-secretory phase, its secretory epithelial cells increase their secretion, which coincides in time with the loss of polarity of the luminal epithelial cells. Cell polarity results from the uneven distribution of intracellular components [19]. Polarity is a fundamental characteristic of all cells and is expressed differently across cell types. Polarity occurs as a result of the action of molecular determinants of polarity, inside the cell, and thus determines both spatial relationships between cells and relationships with the extracellular space [20]. Epithelial cells have apico-basolateral polarity and planar polarity [19]. Thanks to them, a functional barrier is formed, and the orientation of cells in the tissue, the structure of the tissue, intercellular adhesions, and the transmission of signals between cells are regulated. All this also applies to the endometrial epithelium, in which cell polarity defines a repulsive apical surface, governs the movement of molecules around the cell, directs the transmission of signaling molecules between cells, and maintains the integrity of the cytoskeleton [19].

The endometrial luminal epithelial cells first come into contact with the polarized embryonic trophectoderm. They repel each other if the endometrial epithelium maintains its polarity, contributing to an unreceptive state of the endometrium for the attempting implantation blastocyst [2,21]. The endometrial luminal epithelium is no longer polarized during the mid-secretory phase, which makes it receptive [21]. The loss of polarity is due to morphological and molecular changes occurring in the epithelial cells: in the microvilli, the cell membrane surface markers, the intercellular junctions, and the cytoskeletal molecules. To facilitate the invasion, the luminal epithelium weakens the lateral connections between cells by reducing surface epithelial adhesion proteins as well as focal adhesion to the basal membrane [21]. All of these changes can be united under the concept of plasma membrane transformation (PMT), an essential event for implantation to happen [19]. Abnormal endometrial receptivity is one of the main causes of embryo implantation failures and infertility. Any disruption of the PMT affects endometrial receptivity and implantation. Parallels have been drawn between PMT and epithelial–mesenchymal transformation (transition) (EMT) in tumorigenesis [19]. PMT and EMT are similar in the remodeling of the actin cytoskeleton in the attachment zone, integrin expression, and epithelial–stromal communication [19].

### 2.2. Maternal–Placental Circulation and Immunity

In the process of implantation of the blastocyst, the endometrium experiences decidualization. Spiral arteries become less convoluted, with increased diameters. This all increases maternal blood flow to the placenta, and the fetal villi are bathed by the maternal blood that pours into the intervillous space, thus allowing exchange of gases, nutrients, and waste molecules to happen. Fetal chorionic villi contact the maternal blood, without actual fluid exchange. With decreases in the pressure during diastole, the blood with lower oxygen levels and higher carbon dioxide concentration flows back through the endometrial veins [22].

Deoxygenated fetal blood from the fetal circulation passes through the umbilical arteries to the placenta. As they enter the placenta, the umbilical arteries branch radially and form chorionic arteries. The latter branch into cotyledon arteries. In the villi, the smallest vessels form arterial–capillary–venous loops, which bring the fetal blood extremely close to the maternal blood without the intermingling of fetal and maternal blood, thus forming the placental barrier [23]. The placenta serves as a protective barrier for fetal cells, preventing maternal blood cells, proteins, and microorganisms (bacteria and most viruses) from crossing the maternal–fetal barrier [24]. Decline in the placental functioning may lead to the mother-to-child transmission of some infectious diseases [24]. A few viruses, like the rubella virus, the Zika virus, and the cytomegalovirus (CMV), cross the placental barrier, and this happens during specific gestational weeks, when they can progress more favorably because the placenta develops. CMV and Zika viruses come from the maternal blood and cross the barrier to enter the fetal bloodstream [25].

IgG antibodies, which are small in size, can cross the placental barrier after 13 weeks of gestation, with the most considerable number passing in the third trimester, thus providing intrauterine protection to the fetus [26]. This passive immunity serves for several months after birth, providing the newborn with the mother’s long-term humoral immunity to protect the infant through the first months of its life outside the uterus. IgM antibodies have a larger size and cannot cross the placenta [27]. For this reason, infections acquired during pregnancy can be particularly hazardous for the fetus [27].

### 2.3. Hormonal Secretion

As an endocrine organ, the placenta produces HCG (human chorionic gonadotropin), progesterone, estrogen, and HPL (human placental lactogene). With the start of its functioning, the placenta initiates the production of human chorionic gonadotropin (HCG). It stops the cyclicity of menstruation after the corpus luteum ceases activity and atrophies. Its low secretion leads to spontaneous abortion. HCG triggers the production of progesterone and estrogen by the corpus luteum [28]. HCG also helps male fetuses by stimulating the testes to produce testosterone, necessary for the sex organs of the male to grow [28]. Progesterone, like HCG, is essential for the prevention of spontaneous abortion because it stops contractions of the uterus and is vital for implantation [28]. Estrogen assists in the enlargement of the uterus and breasts, sustaining the growth of the fetus and the production of milk. Estrogen has an impact on the vasodilation and thus increases the blood supply during pregnancy [28]. HPL in pregnancy is important for fetal metabolism, general growth, and development. Together with growth hormone, it stimulates insulin-like growth factor production and regulates metabolism [28,29]. HPL modulates embryonic development and metabolism and plays a role in the production of IGF, insulin, surfactant, and adrenocortical hormones in the fetus.

### 2.4. Placental Barrier

The placenta and the fetus may be identified as foreign to the mother’s organism and require protection from the normal immune response that could cause their rejection. The placenta and the fetus are thus treated as immunologically privileged, with immune tolerance. For this, the placenta secretes neurokinin B-containing phosphocholine molecules. These same molecules are cited to be used by parasitic nematodes to deflect detection by the immune system of their host [30]. Small lymphocytic suppressor cells from the fetus can inhibit the response to interleukin-2 and thus inhibit maternal cytotoxic T cells [31]. However, fetal small lymphocytic suppressor cells can be found in the maternal circulation, behind the placental barrier [32].

## 3. Pathophysiology of the Placenta

The impairment of normal placental development is observed in several pathologies of pregnancy: PE, FGR, GTD, and GDM. In addition, environmental factors, such as microorganisms and chemical substances, can influence the placental development and function, unfavorably changing the pregnancy outcome. As a result of these complications, the maternal and fetal mortality and morbidity are increased, which leads to health complications in both mother and child that can remain after pregnancy for life. Maternal diabetes or obesity can change the transportation of nutrients in the placenta, leading to overgrowth or restricted growth of the fetus [33].

## 4. Molecular Approach to Placental Disorders

Assessment of impairment in normal placental development at the molecular level is a current approach in obstetrics research. MiRNAs are small molecules with an immense impact on the regulation of the morphology and functioning of all tissues in the body [34]. Aberrant expression of miRNAs is associated with recurrent spontaneous abortions, PE, FGR, GDM, preterm birth, and small-for-dates [34,35].

MiRNAs are short, non-coding, single-stranded RNAs. They play a role as post-transcriptional modulators of gene expression [36]. Today, over two thousand human miRNAs are known, and every miRNA can target hundreds of genes. Their number is increased by adding their isoforms, which can act cooperatively with the canonical form or have a pretty different function [37,38]. Data have been gathered for the expression of miRNAs in the human placenta at various gestational ages. The identified miRNAs are thought to be essential regulators of placental development and maintenance of pregnancy [34].

### 4.1. Biogenesis and Biological Function of miRNAs

**Figure 1:**
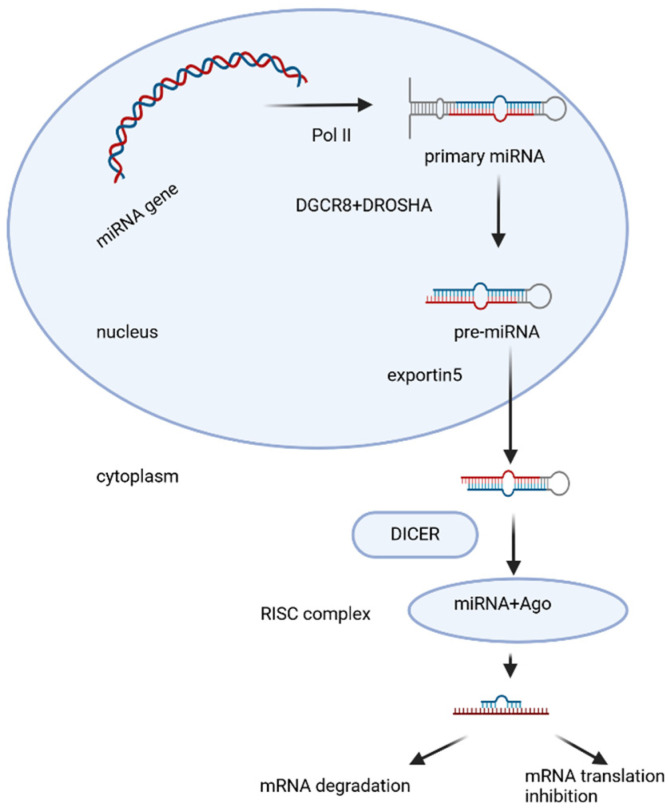
Model of canonical miRNA biogenesis. MIR genes are transcribed as long primary transcripts (pri-miRNAs) by RNA polymerase II. In the nucleus, pri-miRNA undergoes endonuclease cleavage, which is carried out by a microprocessor—a protein complex including the enzyme DROSHA (ribonuclease III) and the dsRNA-binding protein DGCR8 (DiGeorge syndrome critical region 8). The resulting double-stranded precursor (pre-miRNA) is transported across the nuclear membrane to the cytoplasm by the protein Exportin-5. In the cytoplasm, pre-miRNA is further cleaved by the endoribonuclease enzyme DICER. After cleavage of the pre-miRNA, a miRNA duplex is obtained. The miRNA duplex is loaded onto the protein AGO2, a major component of an effector complex, miRISC. The miRNA duplex unfolds. The strand with lower internal stability at the 5′ end is retained in the complex—mature miRNA. The other strand is discarded and degraded. Created with BioRender.com. Nikolova, M. (2025) https://BioRender.com/j5j1zcr (accessed on 14 January 2026).

MiRNAs were discovered in the 1990s, and the majority have been sequenced. There are around 3000 known miRNAs [37]. MiRNAs are encoded mainly by their genes—MIR genes, as well as other types of genomic loci. MIR genes are transcribed as long primary transcripts (pri-miRNAs) by RNA polymerase II [39]. Pri-miRNA contains a partially self-complementary region that folds over to form a hairpin structure (Figure 1) in which a single-stranded loop and a double-stranded (ds) stem are found, terminating in a single-stranded 5′ end and 3′ end. Pri-miRNA undergoes multistep processing to finally form the mature miRNA (Figure 1). The hairpin structure is capped with 7-methylguanosine and is polyadenylated [40]. In the nucleus, pri-miRNA, which is approximately 65–80 nt in length, undergoes endonuclease cleavage, which is carried out by a microprocessor—a protein complex including the enzyme DROSHA (ribonuclease III) and the dsRNA-binding protein DGCR8 (DiGeorge syndrome critical region 8) [41,42,43]. DROSHA cleaves the double-stranded stem 11 nt from the junction with the single-stranded ends. The precision of this cutting is thought to be due to DGCR8, which serves as a kind of molecular meter, recognizing the pri-miRNA substrate and preparing the way for DROSHA [44]. The resulting double-stranded precursor (pre-miRNA) is transported across the nuclear membrane to the cytoplasm by the RanGTPase-dependent protein Exportin-5 (Figure 1). In the cytoplasm, pre-miRNA is further cleaved by the endoribonuclease enzyme DICER, assisted by the transactivation response element RNA-binding protein (TPBP) [44,45,46,47]. DICER (ribonuclease III) is an enzyme with multiple domains, including an amino-terminal PAZ helicase domain (Piwi/Argonaute/Zwille), and two RNase domains [48]. DICER recognizes and captures the free ends of the pre-miRNA and places it in a catalytic enzyme pocket. At this stage, the PAZ domain is important to measure the double-stranded stem from the 3′ end to produce a short dsRNA (miRNA duplex) of species-specific length and a typical two-nucleotide overhang at the 3′ ends [49]. After cleavage of the pre-miRNA, a miRNA duplex of 20–24 nt in length is obtained, with each 3′ end 2 nt longer than the corresponding 5′ end (overhanging 3′ end). It is loaded onto the protein Argonaute2 (AGO2), which is a major component of an effector protein complex called miRISC (miRNA-induced silencing complex) [50,51]. At this stage, the miRNA duplex unfolds, the strand with lower internal stability at the 5′ end is retained in the complex (mature miRNA), and the other strand is discarded and degraded [52,53] (Figure 1). This canonical pathway occurs in most mammals, but alternative biosynthetic pathways have also been described.

The processing of miRNAs to the achievement of mature cognates is affected by the occurrence of changes in the base sequence or the structure of the molecule [36]. The mature miRNAs act on the post-transcriptional level, suppressing the translation of target mRNAs. As a result, their silencing or degradation leads to further downregulation of the target genes. The binding sites for MiRNAs are predominantly found in the 3′UTR of the messenger RNAs (mRNAs). The sequence at the 5′ end, from 2 to 7nt (nucleotide), is vital for the recognition of their targets and has been called the “seed sequence” [34,37,40]. Mature miRNAs mainly bind with the “seed sequence” to the 3′UTR of the corresponding targeted mRNA [37]. The nucleotide sequence in the seed region is essential for correct target recognition. If the binding of miRNA is partially complementary to the 3′UTRs of the mRNA, then the targeting is imperfect and can lead to repression of protein translation. The imperfect binding of miRNAs results in the breakup and degradation of the targeting mRNAs [37]. Some miRNAs can attach to targeting sites at the 5′-untranslated region (5′-UTR). MiRNAs are accepted as negative regulators of gene expression in proliferating cells but upregulate gene expression in latent state cells [40]. Both biogenesis and function of miRNAs are precisely regulated, and any dysregulation of miRNAs leads to human diseases [34].

### 4.2. Expression of miRNAs in Human Placenta

In total, 762 miRNAs have been detected in the human placenta [34]. It has been accepted that hypoxia, mainly resulting from shallow invasion of the trophoblast or some other types of oxidative stress-induced conditions, may deteriorate human placental development and function during pregnancy [54]. It leads to an increased incidence of diseases associated with pregnancy. Hypoxia is an important factor controlling the expression of a number of miRNAs [55]. Hypoxia upregulates miRNA-210 expression (miR-210, which is noted to be a hypoxia sensor) and enhances the processes in the mitochondria in human trophoblast cells [56]. Hypoxia induces upregulation of miR-141, which affects apoptosis, invasion, and vascularization in trophoblast cells [57]. The upregulation of miR-218 caused by the hypoxia suppresses trophoblast cell function [58]. Additionally, other factors, like environmental factors and epigenetic transformation, also impact the expression of miRNAs throughout pregnancy. Some chemical agent influence has been studied as well, namely phthalates, mainly used as plasticizers, added to plastics, to improve their flexibility, transparency, and durability [59]. The maternal exposure to phthalates arouses oxidative stress, and alterations in miRNA expression in the placenta are observed: miR-17-5, miR155-5p, miR126-3p, and miR16, which can finally trigger apoptosis of HTR8/SV neo cells (first-trimester placental cell line) [60]. Exposure to phenol also causes changes in miRNA expression in the placenta, including miR-142-3p, miR-15a-5p, and miR-185 [61]. Analogous to this is the effect on miRNA expression of metals and air toxins, both associated with pregnancy-related complications [62].

Heritable changes can occur as a result of epigenetic regulation in the expression of genes and the expression of human placental miRNAs. DNA methylation has been shown to mediate the expression of miRNAs [63]. An example of this is the C19MC microRNA locus that is imprinted in the human placenta [64]. Methylation leads to downregulation of C19MC—as a result, a larger size of the placenta is observed, which leads to a larger body size of the child [65].

Today, studies have been carried out to prove that numerous miRNAs exert effects on the proliferation, apoptosis, migration, and invasion of the trophoblast cells, thus controlling placental development. In an in vitro study, first-trimester placental explants were used to show that small interfering RNA-mediated knockdown of DICER notably fosters cytotrophoblast cell proliferation. It considerably induced, as well, the expression of two pro-mitogenic signaling molecules—ERK and SHP-2—within the cytotrophoblast [66]. This experiment indicates the role of DICER in the alteration in the expression of miRNAs and its impact on the regulation of human early placental development. Some miRNAs have been shown to affect trophoblast cell proliferation and apoptosis. MiR-376c promotes trophoblast cell proliferation by impairing transforming growth factor-β (TGF-β); miR-210-3p regulates trophoblast cell proliferation by fibroblast growth factor1 (FGF1); miR-133b promotes trophoblast cell proliferation by restricting SGK1 [67,68,69]. Conversely, miR-155 inhibits the proliferation of extravillous trophoblast cells by the downregulation of cyclin D1, miR-424 suppresses trophoblast cell proliferation by regulating ERRγ, and miR-184 promotes apoptosis of trophoblast cells by targeting WIG1 [70,71,72]. The expansion (e.g., the migration and invasion of EVTs (extravillous trophoblasts) into the uterine decidua and myometrium) is essential for effective placentation [73,74]. In EVTs of the human first-trimester placenta, DICER is perceived to be sufficiently expressed [75], thereby proposing that miRNAs have a key role in the migration and invasion of the trophoblast cells. A number of studies reveal that several miRNAs participate in the regulation of migration and invasion of the trophoblast cells. MiR-34a targets MYC, resulting in the inhibition of human trophoblast cell invasion [76]; miR-27a negatively influences the trophoblast cell migration and invasion through mediating SMAD2 [77]; miR-320a negatively regulates trophoblast cell invasion by targeting estrogen-related receptor-gamma [78].

### 4.3. MicroRNAs and Human Gestational Disorders

Abnormal expression levels of miRNAs have been found both in vitro and in vivo in various gestational complications, such as PE, FGR, GTD, and GDM. Several studies comment on the dysregulation of miRNA expression in compromised human pregnancies [8]. We focus our attention on miRNA’s role in preeclampsia, as the data on the topic we found in the literature was the most abundant, and because it could be a life-threatening condition with high clinical importance for both mother and child.

### 4.4. Preeclampsia (PE) and miRNAs

Preeclampsia (PE) is a common disease during pregnancy, which occurs after 20 weeks of pregnancy, comprises changes in multiple organs (causing multiple organ dysfunction, e.g., renal, hepatic, neurologic, hematologic, and others), and is a life-threatening condition for the pregnant woman and the fetus; however, the etiology and pathogenesis of preeclampsia are still inconclusive. PE’s main symptoms are considered to be maternal hypertension and proteinuria [79,80]. The incidence of preeclampsia is 2–8% [81]. The clinical symptoms of most patients with preeclampsia are resolved shortly after a pregnancy is terminated, showing that the placenta plays an essential role in the pathogenesis of preeclampsia [82]. Recently, many studies have been carried out to prove that miRNAs participate in the regulation of both physiological and pathological mechanisms of functioning of the placenta. The expression of miRNAs varies between the placentas from women with PE and normal placentas. The expression of various miRNAs changes in the placentas of women with PE [83,84]. The abnormal expression of those miRNAs in the trophoblast cells arrests their proliferation and causes inadequate invasion, which leads to failure in sufficient remodeling of maternal spiral arteries and possibly a consequent deficiency in angiogenesis, the so-called impaired invasion of the trophoblast [85,86].

**Figure 2:**
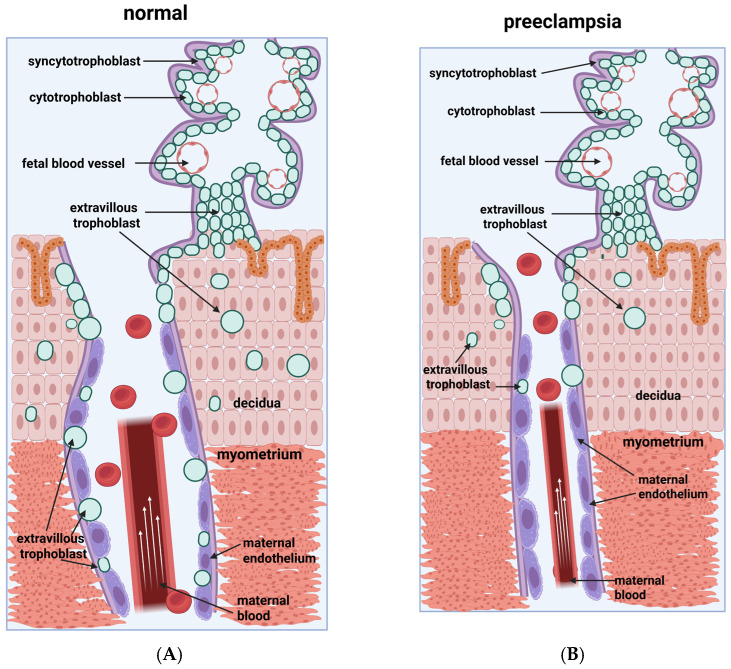
(**A**) The normal placentation is depicted and compared with the preeclamptic version, as shown in (**B**). There is a shallow invasion of the trophoblast in preeclampsia, meaning the replacement of endothelial cells by trophoblast cells is superficial, leading to a lack of maternal vessel relaxation. Created with BioRender.com. Nikolova, M. (2025) https://BioRender.com/iyy86re. Nikolova, M. (2025) https://BioRender.com/pkmw8tq (accessed on 14 January 2026).

One of the essential steps of normal placenta formation is the conversion of uterine spiral arteries into larger, relaxed vessels. The invasion of the trophoblast in the sub-endometrial tissues and the spiral arteries is the primary physiologic mechanism by which this is achieved. The so-called shallow invasion of the trophoblast is a characteristic of preeclampsia, and narrow, unconverted spiral arteries are observed (Figure 2). This leads to hypoxia, which causes endothelial injury and is manifested by hypertension of the mother, proteinuria, and edema.

Trophoblast cells are derived from the trophectoderm of the developing blastocyst and are the first cells to differentiate. The trophoblast adheres to the uterine lining, and implantation starts. Throughout pregnancy, the trophoblast stem cells of the villous cytotrophoblast persist. Yet, the differentiation of trophoblasts into two separate cell lines—the syncytiotrophoblast and the invasive trophoblast—continues until pregnancy ends (Figure 2A).

The placenta plays a key role in the exchange of nutrients and gases and the production of placental hormones and growth factors. These placental functions are mainly carried out by the cells of the syncytiotrophoblast of the chorionic villi. The fetal placental arteries and veins develop within the chorionic villi.

The invasive trophoblast, a second trophoblast lineage, is interstitial or endovascular. At the beginning of pregnancy, the cytotrophoblasts proliferate and form cell columns. From these cell columns, extravillous trophoblasts start and form the endovascular and interstitial invasive trophoblasts. The interstitial invasive trophoblast migrates and invades the uterine tissue, and thus, the connection of the placenta to the uterus is formed. The endovascular invasive trophoblast cells migrate to the uterine spiral arteries.

In the spiral arteries, the trophoblast displaces and replaces the endothelial cell lining and potentiates the degradation of the muscle and elastic coat that are responsible for the vessel integrity. As a result of this loosening of the maternal spiral artery, called conversion, vessels of low resistance and high capacitance are achieved, which meet the necessities for sufficient blood flow to the placenta with the progression of pregnancy. This conversion of the spiral arteries must happen by the end of the first trimester in a healthy pregnancy (Figure 2A).

Defective placentation is thought to be the basis of several diseases associated with pregnancy, including preeclampsia. The significant clinical signs of preeclampsia are maternal edema, pregnancy-induced hypertension, and proteinuria, and in the most severe form, eclampsia, seizures may occur. The etiology of preeclampsia is well known, but its molecular basis is still enigmatic. Shallow trophoblast invasion and failure to convert the spiral arteries are considered basic pathophysiologic mechanisms in the placental pathology of preeclampsia [85] (Figure 2B). In this review, we focus on the molecular mechanisms that can influence the development of preeclampsia.

The miRNAs with significantly upregulated expression in PE placenta, including miR-17 [87], miR-155 [70], miR-431 [88], miR-30a-3p [89], miR-20a [90], miR-20b [91], miR-29b [92], miR-16 [93], miR-675 [94], miR-141 [95], miR-142-3p [96], miR-125b [97], miR-517a/b [98], and miR-517c [99], negatively regulate the proliferation, migration, invasion, and apoptosis of trophoblast cells. MiRNAs’ impact on trophoblast cells depends on their regulation of the target genes. The upregulated expression of miR-17 is proven to have an impact on PE. MiR-17 hints at trophoblast cell function by targeting EPHB4 and ephrin-B2, which are responsible for early placental development [87]. On the other hand, the miRNA suppression of related genes causes alterations in trophoblast cell function, like growth and mobility. MiR-125b (Table 1) inhibits the migration and invasion of extravillous trophoblast by suppressing its target STAT3 expression. STAT3 plays a role in cell infiltration and vascular proliferation [100].

MiR-3935 expression is notably decreased in preeclamptic placentas. It promotes EMT of human trophoblast cells by regulating TRAF6-RGS2 signaling [113] (Table 2). MiRNAs can be packed into placenta-derived exosomes (extracellular vesicles). A differential expression was observed between miRNA species in exosomes from women with PE compared to controls with normal pregnancies [110,111]. The placental exosomes could be considered mediators in the pathogenesis of PE and can find a place in clinical practice as potential biomarkers of PE diagnosis.

Exosomes, derived from the placenta, probably mediate the communication between maternal and fetal cells. A hypothesis has been expressed that these exosomal microRNAs play a role as mediators in cellular processes, e.g., proliferation, apoptosis, invasion, and migration, all dysregulated in PE. In one study, evidence was gathered supporting a downregulation of miR-153-3p, miR-653-5p, and miR-325 in exosomes of a PE group [110]. The following miRNAs, miR-222-3p, miR-486-1-5p, miR-486-2-5p, miR-155, miR-136, miR-494, and miR-495, are upregulated in exosomes in a PE group [109,110,111,112]. In addition, some miRNA SNPs are associated with PE, for example, the polymorphism of miR-152 rs12940701 [114] and miR-146a rs2910164 [115].

**Table 2:** Expression levels (downregulated miRNAs) of miRNAs targeting trophoblast cell migration, proliferation, invasion, apoptosis, and EV (extracellular vesicle) inclusion in PE modified after Jin et al., 2022 [8]. With ↑ we indicated the increase in function, with ↓ we indicated the decrease in function.

Downregulated miRNAs	Affected Biological Process
miR-378a-5p [116], miR-376c [117], miR-335 [118], miR-126 [119]	proliferation ↑
miR-378a-5p [116], miR-3935 [113], miR-376c [117], miR-126 [119]	migration ↑
miR-378a-5p [116], miR-3935 [113]	invasion ↑
miR-335 [118], miR-125a [120]	apoptosis ↓
miR-126 [119]	differentiation ↑
miR-195 [121]	other/targets ↑
miR-153-3p [110], miR-653-5p [110], miR-325 [110]	in exosomes

### 4.5. Preeclampsia and Piwi-Interacting RNAs (piRNAs)

Piwi-interacting RNAs (piRNAs) are another type of small RNA molecules that are cited to play an essential role in the epigenetic regulation of the placenta function in health and disease, especially in PE, as spermatozoa have an important impact on placenta formation [122]. They are the largest class of small non-coding RNA molecules expressed in animal cells; over 20,000 piRNAs are known [123,124]. Piwi-interacting RNAs (piRNAs) are non-coding RNAs that are highly expressed in somatic [122] and germ cells [125] and are associated with fundamental regulations like cell cycle regulation, proliferation, energy metabolism, and immune microenvironment regulation, exerting their role by epigenetic gene suppression and through multiple signaling pathways [126,127]. Emerging evidence demonstrates that piRNAs are observed in several human tissues and are associated with tumorigenesis [122,123,125].

**Figure 3:**
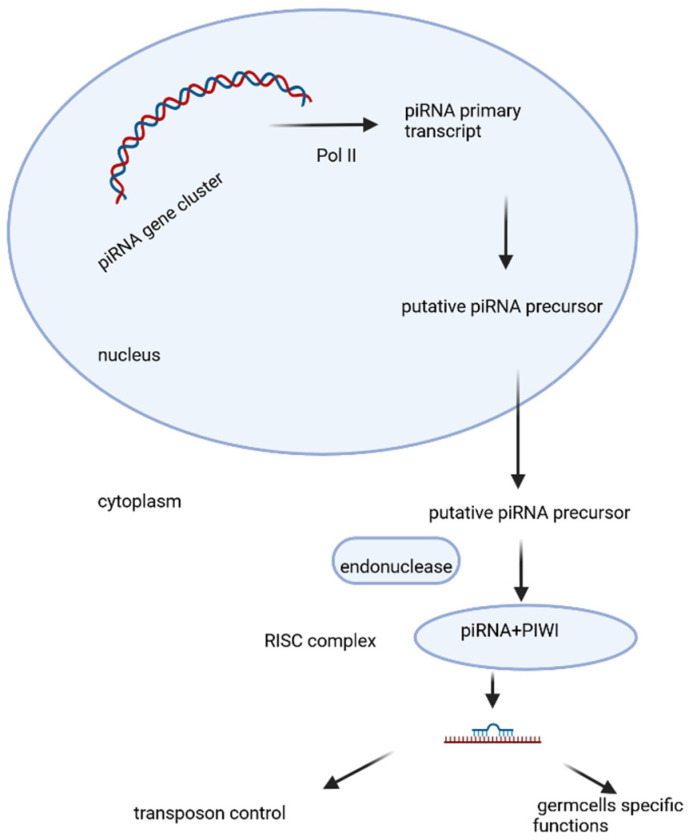
PiRNAs biogenesis, maturation, and biofunction. The piRNA primary transcript is transcribed as a single-stranded RNA by RNA polymerase II from piRNA clusters. The putative precursor matures in the cytoplasm and associates with PIWI proteins in the RISC complex, which is involved in the post-transcriptional silencing of transposable elements (transposons) and functions in the regulation of cellular genes, other false or repeat-derived transcripts. Created with BioRender.com. Nikolova, M. (2025) https://BioRender.com/0stl50d (accessed on 14 January 2026).

PiRNAs are a class of small RNAs that comprise 24–31 nucleotides in length. The precursor is transcribed as single-stranded RNA by RNA polymerase II from genomic regions known as “piRNA clusters” [122]. The putative precursor matures in the cytoplasm and associates with PIWI proteins, an Argonaute subfamily including PIWIL1, PIWIL2, PIWIL3, and PIWIL4. A summary of what is currently known about the biogenesis of piRNA, its potential biological functions, and the possible underlying mechanisms is given in Figure 3 [126]. These piRNA complexes are primarily involved in the post-transcriptional silencing of transposable elements (transposons) and function in the regulation of cellular genes, other false or repeat-derived transcripts (Figure 3). They can participate in the regulation of other genetic molecules in germ-line cells [126,127,128].

In some studies, piRNAs have shown transgenerational inheritance, e.g., they can pass on the memory of “self” and “nonself,” suggesting an impact on various cellular processes that have been identified; however, both the molecular mechanisms through which mature piRNAs are elaborated and the effector points of gene silencing are still partially enigmatic [126].

Changes in piRNAs expression have recently attracted the attention of investigators. In the placenta, the expression of paternal genes is thought to be predominant over the maternal genes [127]. It has been hypothesized that piRNAs in the placenta, which are mainly of paternal origin, can have a more substantial epigenetic influence on gene expression and function of placental cells. PiRNA expression in spermatozoa is significant, and epigenetic influence over spermatogenesis has been studied in detail [129]. PiRNA profiles have been acquired by small RNA sequencing. To identify the differentially expressed piRNA in preeclampsia, their profiles have been compared to those of control patients (healthy controls) [130]. In total, 41 upregulated and 36 downregulated piRNAs were identified in preeclamptic samples. Functional enrichment analysis of the piRNA target genes revealed their influence on extracellular matrix formation and tissue specificity. The expression pattern of the PIWIL-family proteins in the placenta was examined, and PIWIL3 and PIWIL4 were found to be the primary subtypes in the human placenta. Overall, the changes in the expression pattern of piRNA in preeclampsia provide new insights for the regulatory role of piRNA in the human placenta [130].

## 5. Discussion

The investigation of the influence of miRNAs and piRNAs on PE is an evolving field at the intersection of molecular biology and obstetrics. It can provide new, profound insights into the current understanding of the complex pathogenesis of PE. More and more data is being gathered about the expression profiles of non-coding RNAs associated with PE. Yet, there are still gaps and limitations in the literature. There is a lack of consistency across studies because of different gestational ages, different sampling sites from the placenta, and individual patient variations. All of this creates difficulties when considering the development of a set of miRNAs for PE diagnosis in a clinical context. In the future, more profound research on piRNAs and their influence on PE could elucidate paternal impact on PE developmental mechanisms.

## 6. Conclusions

The placenta is a temporary organ that originates from the trophoblast of the embryo and serves for the turnover of nutrients, gases, and waste products between the mother and the fetus. It has certain endocrine functions and plays a role as a preventive barrier for the fetus and can supply the fetus with protective immune factors. Specific abnormalities in its structure and function are detected in several placental disorder conditions, such as preeclampsia, fetal growth restriction, gestational trophoblastic diseases, and gestational diabetes mellitus, whose central pathophysiological mechanism is believed to be realized through hypoxia and oxidative stress. An actual molecular approach for understanding the diseases may serve to find therapeutic solutions for idiopathic placental disorders, like preeclampsia. Small RNAs as epigenetic regulators are promising diagnostic tools. MiRNAs are already being used in some experimental studies as therapeutic molecules. PiRNAs, a class of small RNAs, are highly represented, especially in germ cells, and play a pivotal role as transposon silencers. Having in mind that the trophoblast is primarily of paternal origin, the role of piRNAs in trophoblast formation and functioning is undeniable. Both miRNA and piRNA expression patterns can affect trophoblast cells’ migration, proliferation, invasion, and apoptosis.

With this review, we tried to summarize some of the knowledge about the molecular regulation of impaired invasion of the trophoblast, as in PE. This can aid future studies in further investigating the topic and identifying novel potential applications of miRNAs and piRNAs in the early diagnosis and clinical treatment of preeclampsia.

## Figures and Tables

**Table 1:** Expression levels (upregulated miRNAs) of miRNAs targeting trophoblast cell migration, proliferation, invasion, apoptosis, and EV (extracellular vesicle) inclusion in PE modified after Jin et al., 2022 [8]. With ↑ we indicated the increase in function, with ↓ we indicated the decrease in function.

Upregulated miRNAs	Affected Biological Process
miR-155 [70] miR-20a [96], miR-16-5p [93], miR-142-3p [96], miR-200p-3b [101], miR-137 [102], miR-146a [103]	proliferation ↓
miR-155 [70], miR-431 [88], miR-20b [91], miR-16-5p [93], miR-125b [97], miR-200p-3b [102], miR-137 [102], miR-146a [103]	migration ↓
miR-431 [88], miR-30a-3p [90], miR-20a [104], miR-20b [91], miR-141 [95], miR-142-3p [96], miR-125b [97], miR-146a [103], miR-517a/b [100], miR-517c [100]	invasion ↓
miR-30a-3p [90], miR-29b [92], miR-16-5p [93], miR-200p-3b [101]	apoptosis ↑
miR-17-~92 clusters [105], miR-106a~363 clusters [105]	differentiation ↓
miR17 [87], miR-206 [106], miR-210 [107], miR-202-3p [108], miR-155 [109]	other/targets ↓
miR-222-3p [110], miR-486-1-5p [111], miR-486-2-5p [111], miR-155 [109], miR-136 [112], miR-494 [112], miR-495 [112]	in exosomes

## Data Availability

Publicly available data has been analyzed in this study.
